# Dynamic Range Compression of Thermograms for Assessment of Welded Joint Face Quality

**DOI:** 10.3390/s23041995

**Published:** 2023-02-10

**Authors:** Wojciech Jamrozik, Jacek Górka, Gilmar Ferreira Batalha

**Affiliations:** 1 Department of Fundamentals of Machinery Design, Silesian University of Technology, Konarskiego Str. 18a, 44-100 Gliwice, Poland; 2 Department of Welding Engineering, Silesian University of Technology, Konarskiego Str. 18a, 44-100 Gliwice, Poland; 3 Department of Mechatronics and Mechanical Systems Engineering, Polytechnic School of Engineering of the University of Sao Paulo (USP), São Paulo 05508-900, SP, Brazil

**Keywords:** welding, TIG, thermography, dynamic range compression, image processing, image analysis

## Abstract

Temperature is one of the essential parameters in fusion welding. Typically, an uncooled infrared detector acquires 14-bit data, while a human observer can only distinguish about 128 levels of grey. For IR HDR (high dynamic range) images, one of the main goals of dynamic range compression is to enhance the visibility of low-contrast details. It is an important issue because the temperature span in the cross-section of a welded joint and its length are large. In the paper, global approaches for range compression are investigated, such as algorithms that include pixel transformations, histogram equalization (‘he’) and some of its variants. Additionally, multiscale decomposition methods were investigated. All results are obtained for the sequences of thermograms acquired during the TIG welding of plates made of Inconel 625 superalloy. The process was observed with an uncooled IR camera. The application of compression methods led to the generation of low-dynamic-range (LDR) IR images. The algorithms allowed the preservation of global contrast and enhancement of the visibility of hot details in dark and low-contrast areas. All IR representations of the welded samples were evaluated, and relationships between apparent temperature counted in the pixel-level value and weld-face geometry were revealed. Methods based on wavelet transforms were found to be the most suitable for this type of image; nevertheless, a relatively large local noise was generated.

## 1. Introduction

In modern intelligent factories, there is a large number of various sensors installed to monitor different production processes. This is also the case for welding joints, where current and voltage are the main measured, processed, and analyzed parameters for fusion welding techniques. Additionally, vision devices operating in a wide range of wavelengths are applied. Among them, infrared cameras, also called thermographic cameras, are used to quantify absolute or relative temperature changes on the surface of a workpiece. To produce raw infrared data of the welding process, with a wide/high dynamic range (HDR), high-quality infrared cameras accommodate a temperature range of about 800 K (calibration dependent). The thermal detector can catch the temperature difference within 0.01 K. Normally, an uncooled infrared detector acquires 14-bit data, a relatively high dynamic range. At the same time, a human observer can only distinguish about 128 levels of grey in an image. Moreover, the human visual system interprets light nonlinearly: for example, by turning on a second identical light source, the light source is double the brightness, and the difference in perception does not make things seem twice as bright. In addition, humans are much better at distinguishing subtle differences in midtones (grey) than significant differences in bright ones. This is part of the way we can deal with the high dynamic ranges of the scenes we encounter, and digital sensors differ in this respect: doubling the amount of radiation and the number of electrons released by the sensor, results in double the value generated by the A/D converter. Perception is even more challenging when dealing with IR images because the greyscale level or color is not directly connected with a visual property known from experience by the human vision system. Another issue is bounded to the hardware used to present visual content. A standard display device can show an image with only 256 levels of grey (8 bits). The IR image is often transformed to such a range before image processing, and analysis methods are applied. The problem of compression and visualization of HDR images has been extensively investigated, and several visualization techniques have been proposed in the literature. A vast number of elaborated techniques and methods are suitable to deal with visible light images represented in RGB or HSV color space. Those methods are primarily focused on contrast-enhancement-based methods. However, only a few studies have explicitly investigated HDR infrared images [1,2], while thermal analysis is typical for welding monitoring and simulation [3,4] and other mechanical engineering tasks [5]. For IR HDR images, one of the main goals of dynamic range compression, in other words, to obtain a low-dynamic-range (LDR) image, is to enhance the visibility of low thermal contrast details. It is an important issue because the temperature span in a cross-section of a welded joint and its length can be considerable. Therefore, it is not easy to unanimously assess different weld areas, such as the weld face, heat-affected zone, and fusion line. Moreover, any inclusions, pores, spatters and other welding inconsistencies and faults that can be represented in an IR image have temperatures similar to the temperature of surrounding pixels.

The main drawback of HDR images is not only the difficulty of presentation on common types of display but also memory and bandwidth requirements. For those reasons, a method for HDR to LDR compression is definitely required. The general solution for this task is to apply a tone-mapping curve (TMC) or tone-mapping operator (TMO). After that, any LDR device can display the tone-mapped version of HDR images [6]. The most straightforward method is to linearly scale HDR values to fit them into a particular range (e.g.,〈0;1〉). After scaling, relative contrast is preserved, but because of quantisation, the image can lose detail visibility. The main advantage of TRCs lies in their simplicity and computational efficiency. However, these global maps must be monolithic and unchanging to avoid a reversal of the local edge contrast. Therefore, it is fundamentally difficult to retain local contrasts in images where the intensity of the interest regions occupies the whole dynamic range more or less uniformly. Other methods can be applied to preserve IR image details and enhance the readability of temperature distribution patterns. The first group of algorithms aims to reshape the image histogram while emphasizing the most effective pixels, maintaining the dependence between intensity/temperature levels. The most common approach is the application of a histogram equalization algorithm (‘he’).

Histogram equalization is a simple global algorithm that can sometimes be very effective. On the other side, it can produce results that can be too severe. It is because it cannot deal with the local brightness of the processed image. Because of that, deterioration of background contrast and the removal of small objects can occur. To generate results that are not burdened with undesirable effects of the ‘he’ algorithm, an adaptative histogram equalization (‘ahe’) algorithm can be used. To account for the local brightness feature, the whole image is divided into small, overlapped regions, in which the histograms are equalized. This operation led to the achievement of images with stretched contrast. On the other hand, ‘ahe’ can give images affected with noise because of unwanted enhancement in homogenous regions.

In histogram equalization methods, the contrast can be over-enhanced because the amplification is in each region rounded with the slope of surrounding pixels’ CDF (cumulative distribution function). To avoid this, contrast limiting is applied. In contrast-limited ‘ahe’ (‘clahe’ [7]), if the histogram bin exceeds a specific limit, those pixels’ values above the limit are redistributed to other bins before equalization. It leads to the limitation of the CDF slope. The main inconvenience when using ‘clahe’ is the need to select the clipping limit, which is not always a straightforward task. There are also other modifications of the ‘he’ method. In bi- or multi-histogram equalization, there are multiple histograms equalized because the histogram is divided into smaller sub-histograms, and each of them is then processed individually [8,9]. In [10], the adaptive gamma correction method with weighting distribution (‘agcwd’) is proposed. In this approach, the luminance PSD (probability density function) and the correction of gamma (a method allowing the control of the brightness of an image) are used.

Dynamic histogram equalization [11] (‘dhe’) partitions is another modification of the ‘he’ algorithm. In this method, the average brightness of the image is maintained by dividing the histogram into several segments based on local minima. Each part is individually equalized. The equalization of the dynamic histogram that preserves brightness [12] (‘bpdhe’) utilizes Gaussian kernels to smooth the histogram image. Another approach that does not demand any histogram smoothing is the dynamic fuzzy histogram equalization (‘bpdhfe’) [13]. As the fuzzy representation is always smooth, according to fuzzy statistics, grey-level values are remapped between the extremes. Another variation and enhancement method of the ‘he’ algorithm is equalizing the nonparametric modified histogram [14] (‘nmhe’). This algorithm removes, cuts, and normalizes spikes from the input histogram. Then, the sum deviation of this modified intermediate histogram is calculated to build a uniform histogram. Using the CDF of this modified histogram as the transfer function, the image’s contras are enhanced.

Another group of techniques suitable to compress the dynamic range of IR images is based on a multiscale decomposition of images and then merging the intermediate images onto various decomposition stages using appropriate aggregation rules. The decomposition can be made in a spatial domain, but also with a frequency domain transform or wavelet transform. In general, in this method, the result is generated in a two-stage process [15]. Firstly, a set of ranges of additional images (secondary images) is generated based on a single radiometric IR image (primary image). The next step is to merge input images with a proper image aggregation method. The compressed final image is the result of the fusion procedure. Details that were partially hidden or not sharp enough in the primary images have improved visibility and enhanced contrast in the final image. Besides image merging, one of the critical operations is the generation of secondary images. It is completed by a gradual change in input image: from lower to higher values and from higher to lower ones. After this operation, two sets of secondary images are extracted: low (LSSI) and high (HSSI). The number of secondary images and the weighting function steering the image generation task are the procedure’s parameters.

The joining of secondary images is performed using the wavelet transform. Secondary images were merged, taking the mean of the approximations and the minimum/maximum/to mean for the details. The obtained low and high images are combined using the mean for approximation and details. Thus, ‘aamima’ stands for merging details of the LSSI set with the minimum operator, while the HSSI images were combined with the maximum operator. The ‘aamami’ method is the opposite, while ‘aame’ means that the details of both sets were merged using a mean operator [15]. The main drawback of the method is the high dependence of the results on the selection of the weighting function and wavelet used for the result merging. A decomposition–fusion framework eliminates the contradiction between contrast and noise [16]. The input image is rescaled, and it undergoes bilateral feature enhancement. It leads to noise suppression and contrast enhancement through high- and low-frequency feature remapping. Those features are used in a fusion process to reconstruct optimized output images.

There is also a different set of methods that are elaborated and based on Retinex theory and address the problem that human visual systems (HVSs) can separate the reflectance of objects from the intensity of a given scene. It led to efficient algorithms enhancing local image contrast, thus permitting the enhancement of low-contrast areas, color correction and color restoration [17].

Another group of methods appeared last time to improve the results obtained using classic statistical methods. Incorporating machine-learning (ML) techniques, especially deep learning, CNN (convolution neural network) model were trained to allow the processing of noisy images to obtain high-dynamic-range infrared image compression. Moreover, to assure a significant detail-enhancement performance. A clean image is achieved by removing noise from the original image. The noise level to subtract from the image is generated by the neural structure [18].

This paper presents a comprehensive analysis of various dynamic-range-compression methods that can be used to improve the weld-face quality assessment process. Global approaches were investigated, such as algorithms that include fundamental pixel transformations, histogram equalization (‘he’) and some of its variants, multiscale decomposition methods, and Retinex (‘ret’)-based techniques. All results were obtained for sequences of thermograms acquired during the TIG welding of thin plates made from Inconel 625 superalloy. The process was observed with an uncooled FLIR A655sc camera. There was a set of joints made with different parameters. Because of that, joints of other characteristics were obtained. The application of compression methods led to the generation of LDR IR images, and algorithms allowed the image to be reproduced in different scenarios, preserve the global contrast, enhance the visibility of hot objects and details in dark and low-contrast areas, and create gradient reversal, hazy, and saturation artefacts.

## 2. Materials and Methods

Nickel superalloy type Inconel 625 was used as a base material for the test samples that underwent TIG welding. All sheets were 1 mm thick. Material was delivered by Huntington Alloys Corporation (USA). To manufacture the material, an industrial process that involved the smelting of Inconel 625 in a vacuum furnace was performed. After smelting, plastic processing was applied by cold rolling with intermediate heat treatment (recrystallization annealing). The chemical composition of the used material is gathered in Table 1.

**Figure 1:**
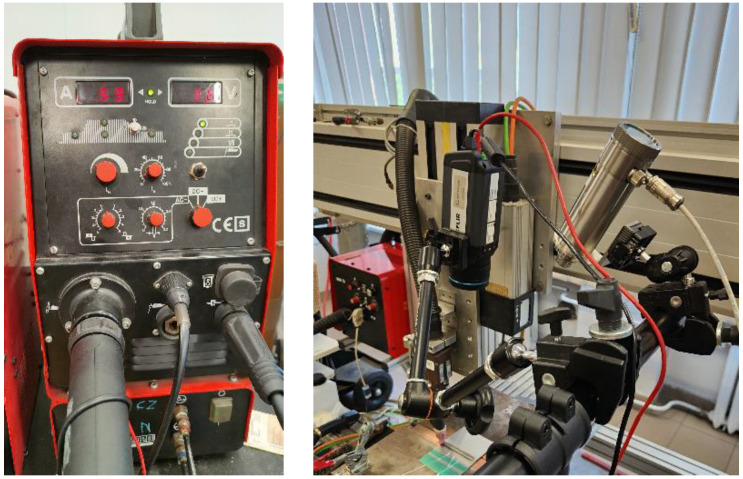
Welding device, TIG torch, and IR camera used during studies.

All workpieces were joined using the Casto TIG 2002 device (Figure 1). TIG welding of thin sheets was conducted under laboratory conditions with the following constant parameters: shield gas flow of Ar 12 L/min and ridge shield gas flow Ar 3 L/min. For welding, a tungsten electrode (thoriated), type WT20 (diameter of 2.4 mm), was used.

Measurement of the temperature distribution in the welded joints was made with a FILR A655sc infrared camera (Figure 1). The camera’s spatial resolution was 640 × 480 px, the dynamic depth was 16 bit, and the emissivity was set at a constant ε = 0.13. Variation in emissivity as a function of temperature was not taken into consideration because, in the proposed methods, there is no need to compare temperatures between the molten and solidified regions of the joint. Moreover, the reflections coming from a hot welding torch and the process itself (welding arc) were compensated only with a constant value of reflected temperature. The IR images were taken at 60 fps. The camera’s optical axis was inclined to the plane of the sample at an angle of 87 degrees, and the distance between the camera and the welded piece was 600 mm.

**Figure 2:**
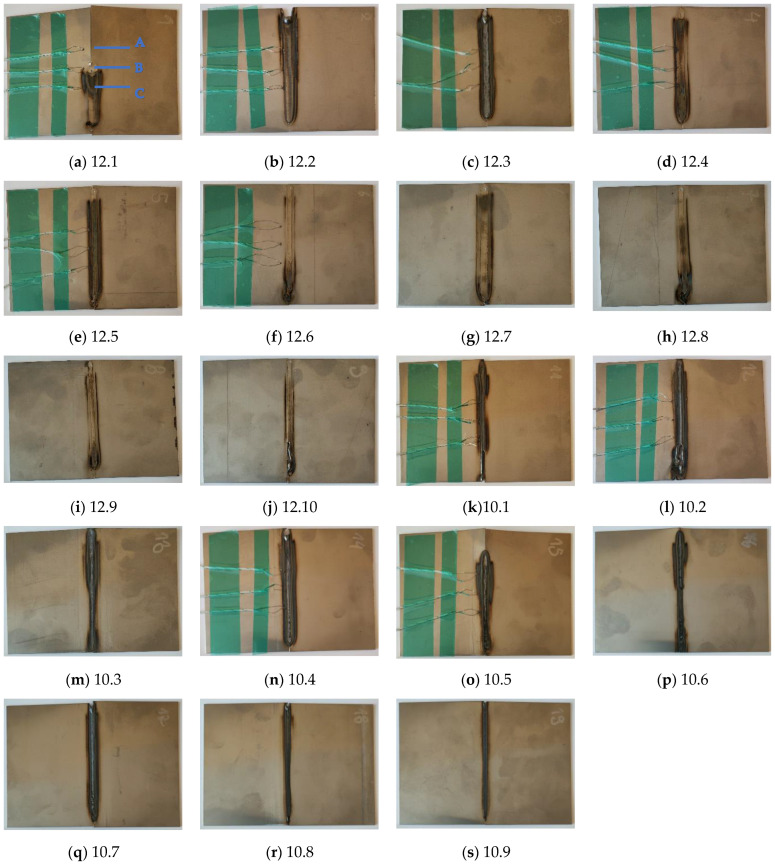
Weld faces made on Inconel 625 sheets. In (**a**), the locations (marked as A, B and C) of the measurements of the width of the weld face are included.

Joints were made for various sets of process parameters collected in Table 2. After all test trials (the samples are presented in Figure 2), the width of the weld face was measured. Measurements were taken at points A, B, and C (Figure 2a), which correspond to the locations of thermocouples. The values were collected in Table 2.

For the objective quality assessment of thermograms, several measures using reference images and the objective approach (without reference images) were considered. There are several image quality measurement methods, considering informational content, focus, sharpness, and other image properties. In gradient and Laplacian-based operators, the key idea is to measure the number of sharp edges in the image. Wavelet and other measures that use multiscale transforms are used to describe an image’s frequency and spatial content. Additionally, statistical measures are often used to assess an image’s spatial content and pixel value distribution. The following measures were used: grey-level local variance (GLLV) [19], histogram entropy (HISE) [20], Tenengrad variance (TENV), the energy of gradient (GRAE), Brenner focus measure (BREN) [19], Tenegrad (TENG) [21], steerable filters-based metrics (SFIL) [22] and histogram range (HISR) [23]. The structural similarity index (SSIM) [24] was also calculated. These metrics are based on comparisons of local luminance (temperature), contrast, and structure between the reference image and the fused image evaluated. The SSIM computation was carried out on a local window by dividing the whole image into N × N size image blocks. For two images, the SSIM is defined as:

(1) $$\mathrm{SSIM}=\frac{\left(2\mu_{x}\mu_{y}+C_{1}\right)\left(2\sigma_{xy}+C_{2}\right)}{\left(\mu_{x}^{2}+\mu_{y}^{2}+C_{1}\right)\left(\sigma_{x}^{2}+\sigma_{y}^{2}+C_{2}\right)},$$

where $\mu_{x}$, $\mu_{y}$ are the mean values, $\sigma_{x}$, $\sigma_{y}$ are variance, $\sigma_{xy}$ is covariance and $C_{1}$, $C_{2}$ are small constants. Another metric used for the assessment of images with compressed dynamic range was the universal image quality index Q  [25], which is defined as follows:

(2) $$Q=\frac{4\sigma_{xy}\mu_{x}\mu_{y}}{\left(\mu_{x}^{2}+\mu_{y}^{2}\right)\left(\sigma_{x}^{2}+\sigma_{y}^{2}\right)},$$

where $\mu_{x}$, $\mu_{y}$ are the mean values, $\sigma_{x}$, $\sigma_{y}$ are variance, and $\sigma_{xy}$ is the covariance between considered images x and y. For all measures, a higher score means better image quality, or the evaluated image is more similar to the original image (for methods requiring a reference image). Moreover, Perception Image Quality Evaluator (PIQE), a no-reference image quality score [26] and Naturalness Image Quality Evaluator (NIQE), another no-reference image quality score [27], were applied to estimate obtained thermogram quality.

**Figure 3:**
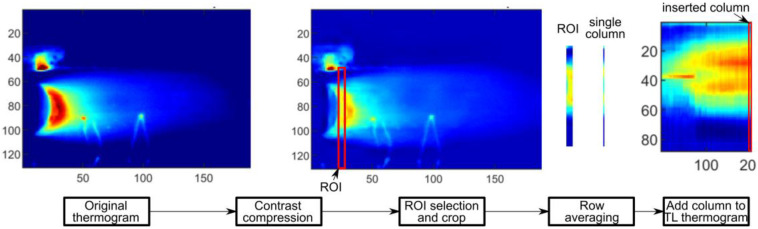
The idea of time–location transformation for thermogram reconstruction.

The reconstruction of a temperature pattern in a joint was made using a time–location (TL) transformation. A region of interest (ROI) placed directly in front of the welding (Figure 3) was selected for a known and fixed position. The width of the ROI was 3 pixels to average the temperature and to avoid random disturbances and small hot spots. Consecutive ROIs were averaged, and the resulting single columns were combined in a 2D matrix, representing the temperature distribution of the entire welded sample at a fixed time moment in a desired area. The TL-reconstructed thermogram can be regarded as an online time-invariant thermogram, where temperatures at the whole seam length were taken in the same micro-time of the process.

**Figure 4:**
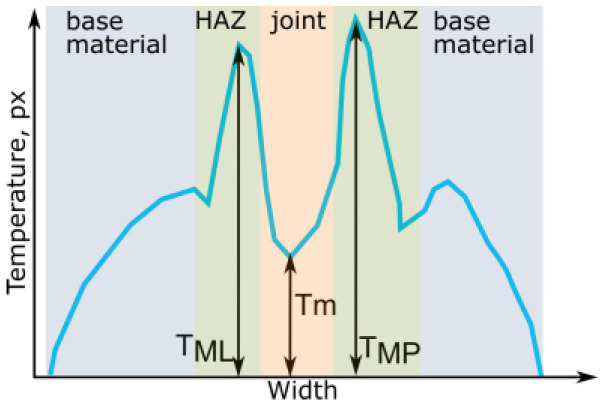
Apparent temperature changes in the cross-section of joint.

The TL-reconstructed thermograms underwent an assessment using a topological feature describing the shape of the vertical cross-section of the temperature profile. The key assumption of that metric used for the considered samples is the presence of a zone characterized with radiational properties than the joint and base material. The width of this zone is related to the presence of the heat-affected zone, where the heat introduced by the welding arc influenced changes in the structure and properties of the base material. When evaluating the samples made (Figure 2), it can be seen that there is a dark area of different widths on both sides of the joint in each sample. This zone should be visible in the IR image (thermogram). In contrast, the emissivity of this zone will be different from the emissivity of the base material (which has a metallic, highly reflective surface characterized by very low emissivity). Additionally, the emissivity of the join will also be lower than in this transition zone. Emissivity variations will lead to changes in apparent temperature on the joint cross-section. The expected temperature distribution at the vertical cross-section is presented in Figure 4.

It can be seen in Figure 4 that the temperature in the joint and in the base material where the heat did not affect the microstructure is lower than in the transition zones. The difference in a raw thermogram will depend only on the difference in emissivity between consecutive areas. However, when a large amount of heat is introduced to the base material, slight differences in emissivity and high radiation will lead to the situation where temperature peaks in the HAZ zone are vague and faint. Mapping temperature values to a color map can lead to an even smaller eminence of temperature peaks. The application of dynamic range compression should emphasize high-temperature areas and make minor differences in temperature described by image pixel values more distinctive. To quantify those temperature differences, a simple measure was introduced:

(3) $${\hat{T}}_{p-p}=\frac{T_{ML}+T_{MP}}{2}-T_{m},$$

where: $T_{ML}$ and $T_{MP}$ are maximal temperature values of the left and right peaks, respectively, $T_{m}$ is the minimal value of the temperature that can be found between the locations of maximal peaks $T_{ML}$ and $T_{MP}$. The temperature, in this case, is reflected by a pixel-level value ranging from 0 to 255. Results of ${\hat{T}}_{p-p}$ were normalized to the range 〈0;1〉.

**Figure 5:**
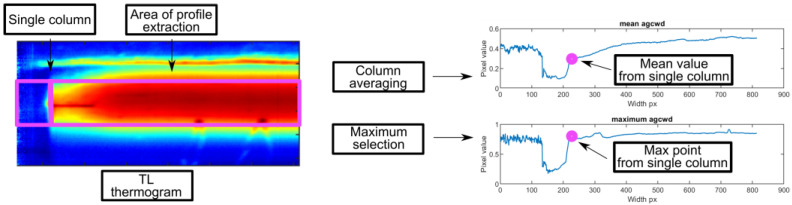
Visualization of weld mean and max profiles calculation.

Another approach used to express the properties and quality of the weld face was based on the extraction of apparent temperature profiles along the seam. For TL thermograms, a column with a center in the joint axis and a length of 6 mm in absolute units was selected. The mean value was calculated along this column, and the maximum was found. Each estimated value was added to a vector that forms a horizontal profile representing temperature changes along the joint (Figure 5).

## 3. Results and Discussion

All sequences considered were transformed using all methods. After preliminary evaluation, several sequences were deemed unsuitable for further investigation; due to the vibrations of the welding stand and IR camera holder, some parts of the acquired sequences were slightly out of focus. Accordingly, only a subset of 14 sequences was further processed and analyzed.

### Compression of Thermograms

**Figure 6:**
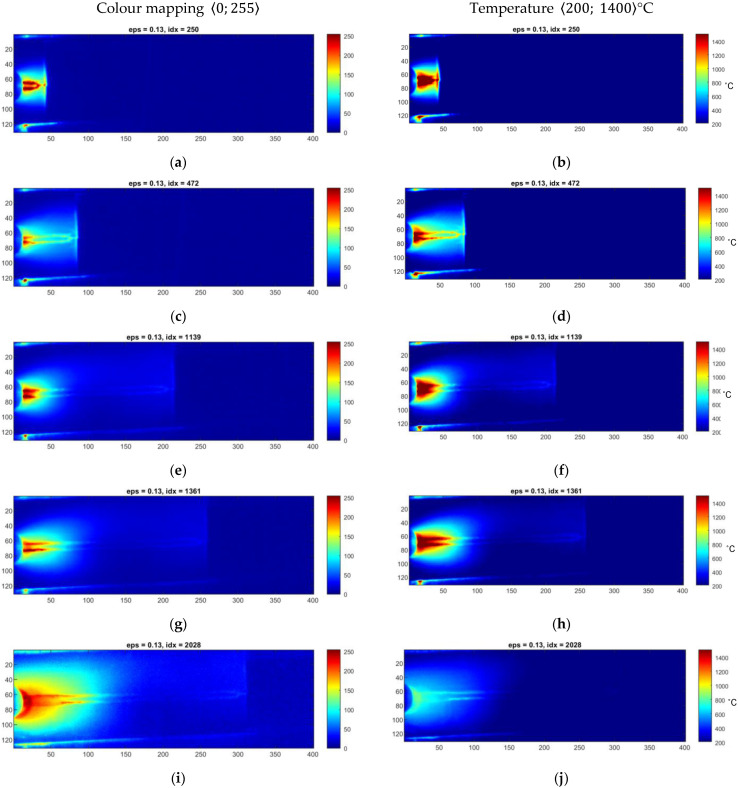
(**a**–**j**) Thermograms made during welding of 1.0 mm thick Inconel 625 sheets (sample ID 10.3).

**Figure 7:**
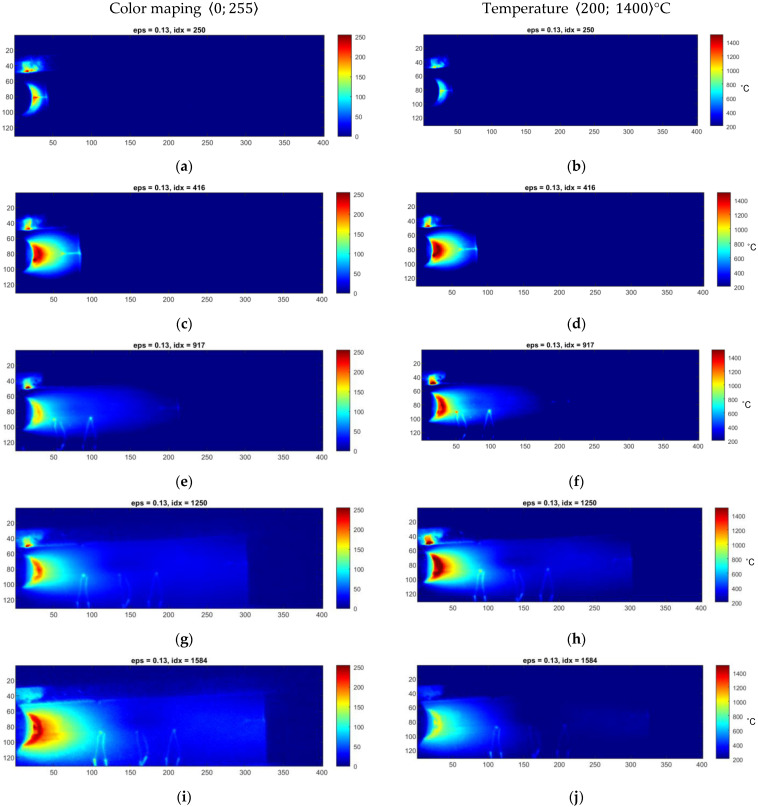
(**a**–**j**) Thermograms made during welding of 1.2 mm thick Inconel 625 sheets (sample ID 12.5).

Raw thermograms from sequences covering exemplary welds made on a metal sheet with a 1.0 mm thickness (Figure 6) and 1.2 mm thickness (Figure 7). In both figures, there are original thermograms (right column) as well as thermograms that are mapped to the 8-bit value space, using a simple linear quantization method, where each pixel can take values from 0 to 255. This mapping type can be regarded as a simple normalization of a thermogram, representing a specific range of temperatures as real numbers covering some of a range. It is the simplest way to reduce the dynamic range of a thermogram. 

It can be seen that the relatively large span of temperature values that is in the range 〈200–1400〉 °C makes the perception of small temperature changes impossible due to the similarity of colours in the chosen colour palette. From the welding quality assessment that should be driven in the online mode, the critical issue is to have visible characteristic zones of a join, heat-affected zone and transition zones. It can lead to an assessment of the joint’s geometry, namely the face or the ridge. Additionally, a statistical evaluation of joints can provide information about joint quality, where uniformity of the joint area with simple statistical features can describe a specific symptom of joint high quality.

In Figure 6 and Figure 7, there are two exemplary sets taken from two sequences taken for different processes realized on Inconel 625 sheets that differed in thickness. Heat input Q, kJ/mm, is the amount of arc energy that reaches the welding workpiece using a certain welding method:

(4) $$Q=\eta \frac{I\cdot U}{1000v},$$

where U is the voltage used in volts (V), I is the current used in amperes (A), v is the travel speed of the welding torch in mm/s and η is the thermal process efficiency. The heat input in both cases is mainly influenced by the welding current and speed, while the change in arc voltage is similar for both cases (*U* 11 V). According to that, for the TIG method, where thermal efficiency *η* = 0.6, the heat input for sample 10.3 is $Q_{10.3}=0.1\;\mathrm{kJ}/\mathrm{mm}$ and for 12.5 $Q_{12.5}=0.08\;\mathrm{kJ}/\mathrm{mm}$. Thus, the amount of heat used for welding is higher for the thinner workpiece. That is why the differences between the weld and base material temperatures are also higher.

**Figure 8:**
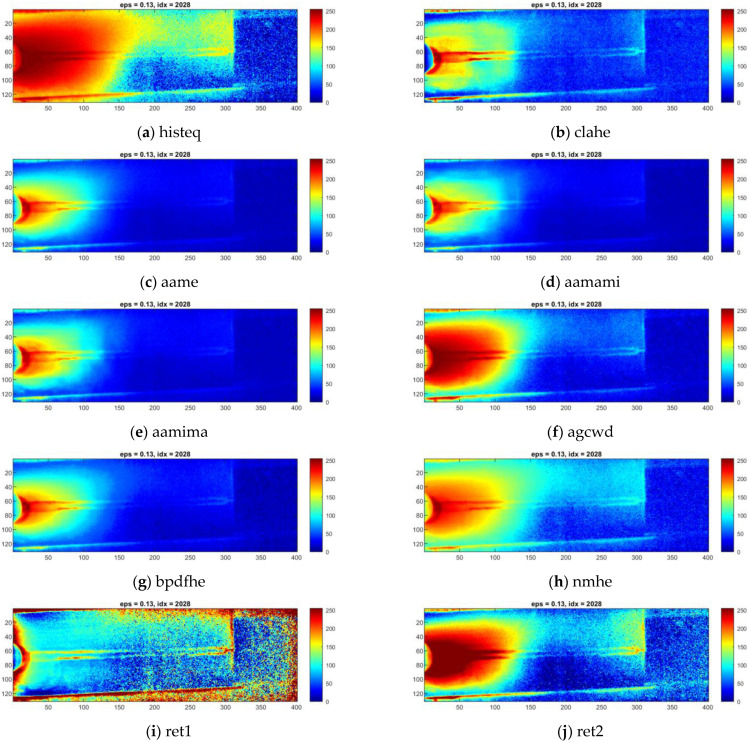
Results of the exemplary thermogram idx = 2028 made during welding of 1.0 mm thick Inconel 625 sheets (sample ID 10.3) enhanced with different compression methods.

**Figure 9:**
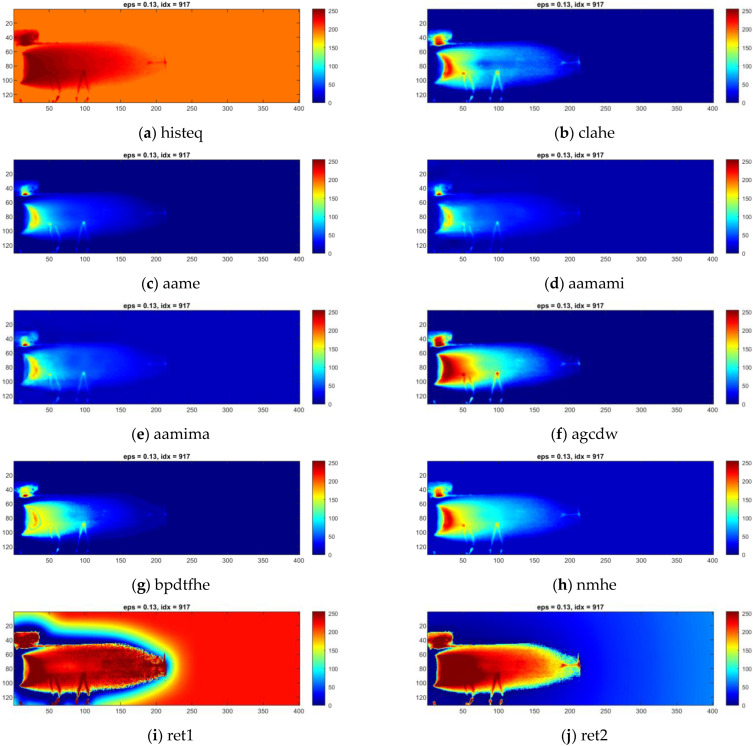
Results of the exemplary thermogram idx = 917 made during welding of 1.2 mm thick Inconel 625 sheets (sample ID 12.5) enhanced with different compression methods.

Individual thermograms in all sequences underwent dynamic contrast processing. Ten methods were used for this task. Exemplary results obtained for a single IR frame are presented in Figure 8 for sample ID 10.3 and Figure 9 for sample ID 12.5. It can be observed that the results produced by the individual methods differ in terms of visibility and the reduction of separability and noise (image smoothing). In Figure 8, for sample ID 10.3, two main zones are distinguishable. First is the so-called hot zone, where the influence of welding arc and reflection from the welding torch dominate. As the most interesting part of this area is close to the welding torch and welding pool, the desired outcome is the highlighted seam, while the hot background is dumped. In the second area, the cold one, the weld and HAZ are barely visible. Thus, the main goal is to increase the pixel value (apparent temperature of the seam) to gain a better contrast between the weld and the parent material. The selectivity of each range compression method is different. For ‘histeq’, ‘agcwd’ and ‘ret2′, the weld in the hot zone is wholly covered with a high background temperature. However, the result given by the ‘ret1′ method allowed the best separation of the weld and HAZ areas, but the amplitude of the point noise was the highest. An analysis of results considering the ID 12.5 sample can lead to other conclusions. In this case, the best selectivity in the hot zone was ensured by wavelet-transform-based methods, namely ‘aamima’ and ‘aamami’. The joint is only partially visible in the cold zone at the beginning, where the temperature was relatively high, on thermograms generated using the ‘ret1′ method. An unequivocal selection of the best, most robust and universal dynamic range compression method is impossible at this stage.

**Figure 10:**
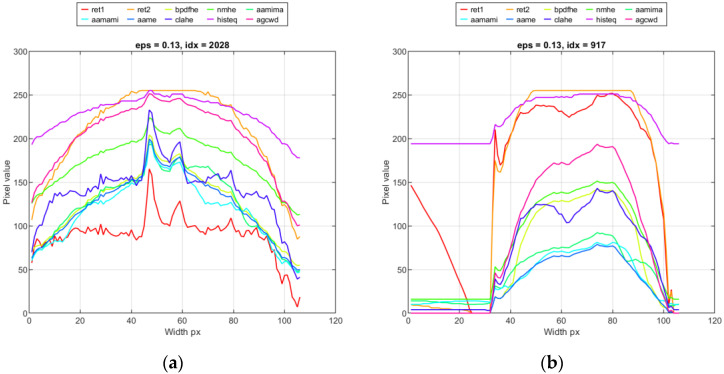
Exemplary vertical profiles obtained by applying range compression methods for samples: (**a**) idx = 2028 ID 10.3, (**b**) idx = 917 ID 12.5.

The application of various algorithms for dynamic range compression leads to different results, thus in-depth analysis is demanded. In Figure 10, a comparison of vertical (column) averaged profiles is presented for certain time frames. It can be seen that the profiles obtained by the ‘ret2′ method are over-saturated. Similar results are obtained using ‘histeq’. Still, in this case, the profile is smoothed, and the characteristic gradients result from different radiational properties of the weld/HAZ zone removed. There are also noticeable differences in the eminence of the HAZ zone and the slope of temperature drop when moving away from the weld. For ID 10.3, the temperature in the parent material is nearly constant for ‘ret1′ and ‘clahe’, while for ‘agcwd’, there is a high temperature drop.

**Figure 11:**
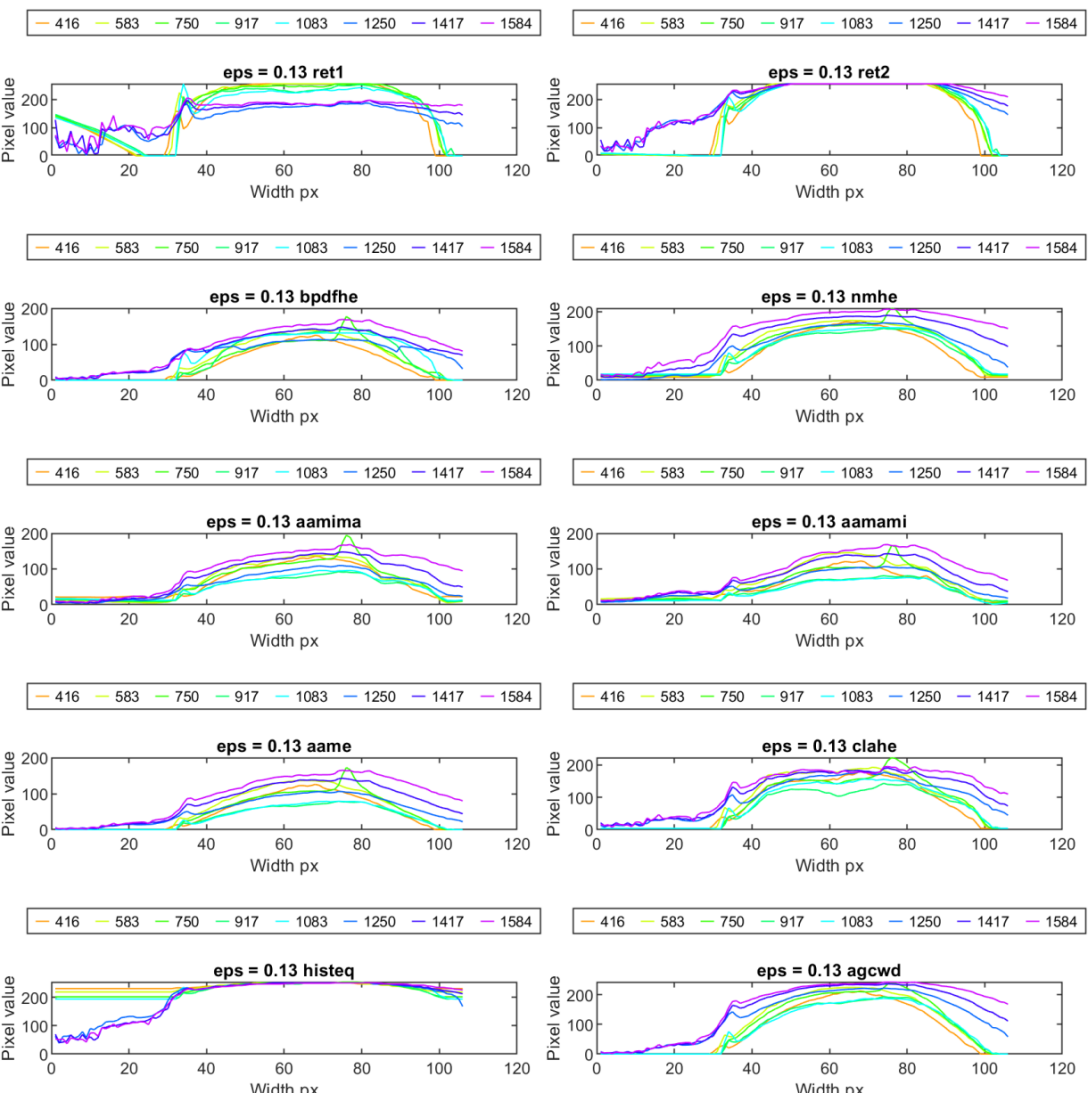
Exemplary vertical profiles obtained by applying range compression methods for samples of chosen frames (idx numbers) in sequence, taken during the welding of the ID 12.5 sample.

When estimating temperature changes during the welding process, the influence of the amount of heat transferred to the weld over time is significant (Figure 11). It is especially noticeable at the beginning of the process because there was a slight delay between the welding arc’s glow and the torch moving along the desired trajectory. When the welding was complete, the end point of the joint remained relatively hot. Moreover, there were reflections present from the hot welding torch. The main drawback of the ‘histeq’ or ‘agcwd’ method is the flattering of small disturbances that lead to the removal of valid informational content that can provide valuable information (being a symptom) of unwanted changes in the process condition that can cause a decrease in the joint and weld face.

**Figure 12:**
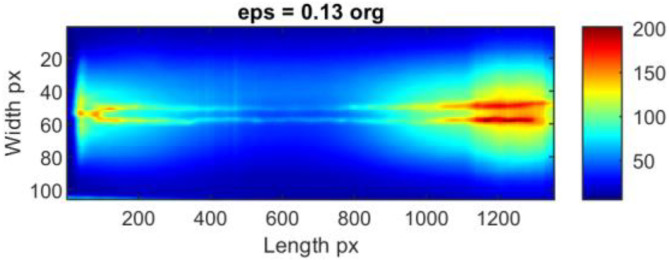
TL-reconstructed thermograms obtained for raw IR images taken during welding of 1.0 mm thick Inconel 625 sheets (sample ID 10.3).

**Figure 13:**
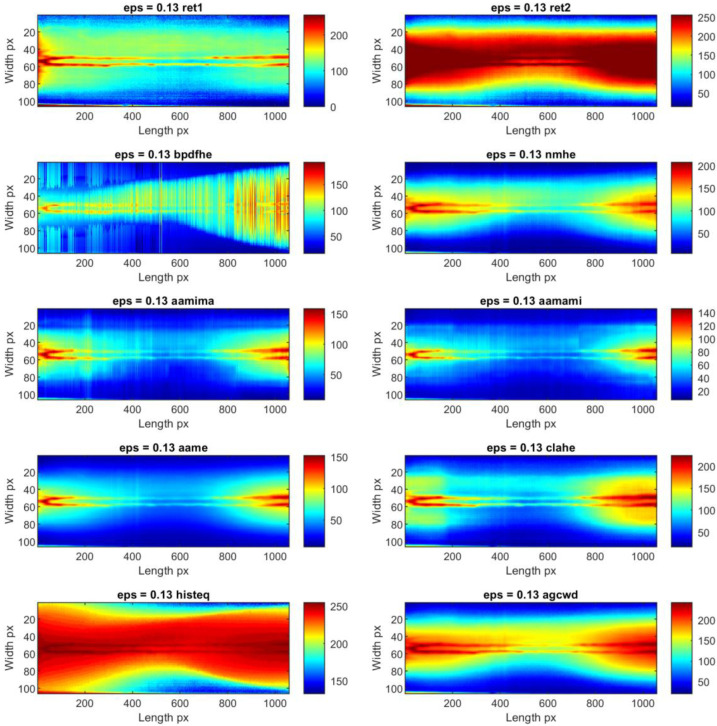
TL-reconstructed thermograms obtained for IR images taken during welding of 1.0 mm thick Inconel 625 sheets (sample ID 10.3) processed with different dynamic range compression methods.

**Figure 14:**
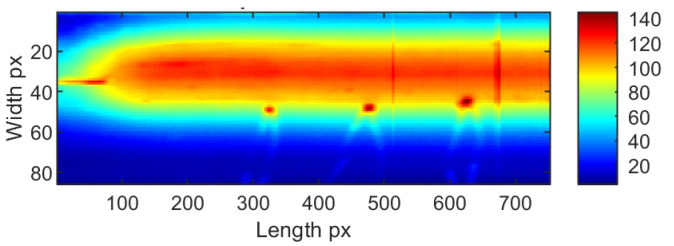
TL-reconstructed thermograms obtained for raw IR images taken during welding of 1.2 mm thick Inconel 625 sheets (sample ID 12.5).

**Figure 15:**
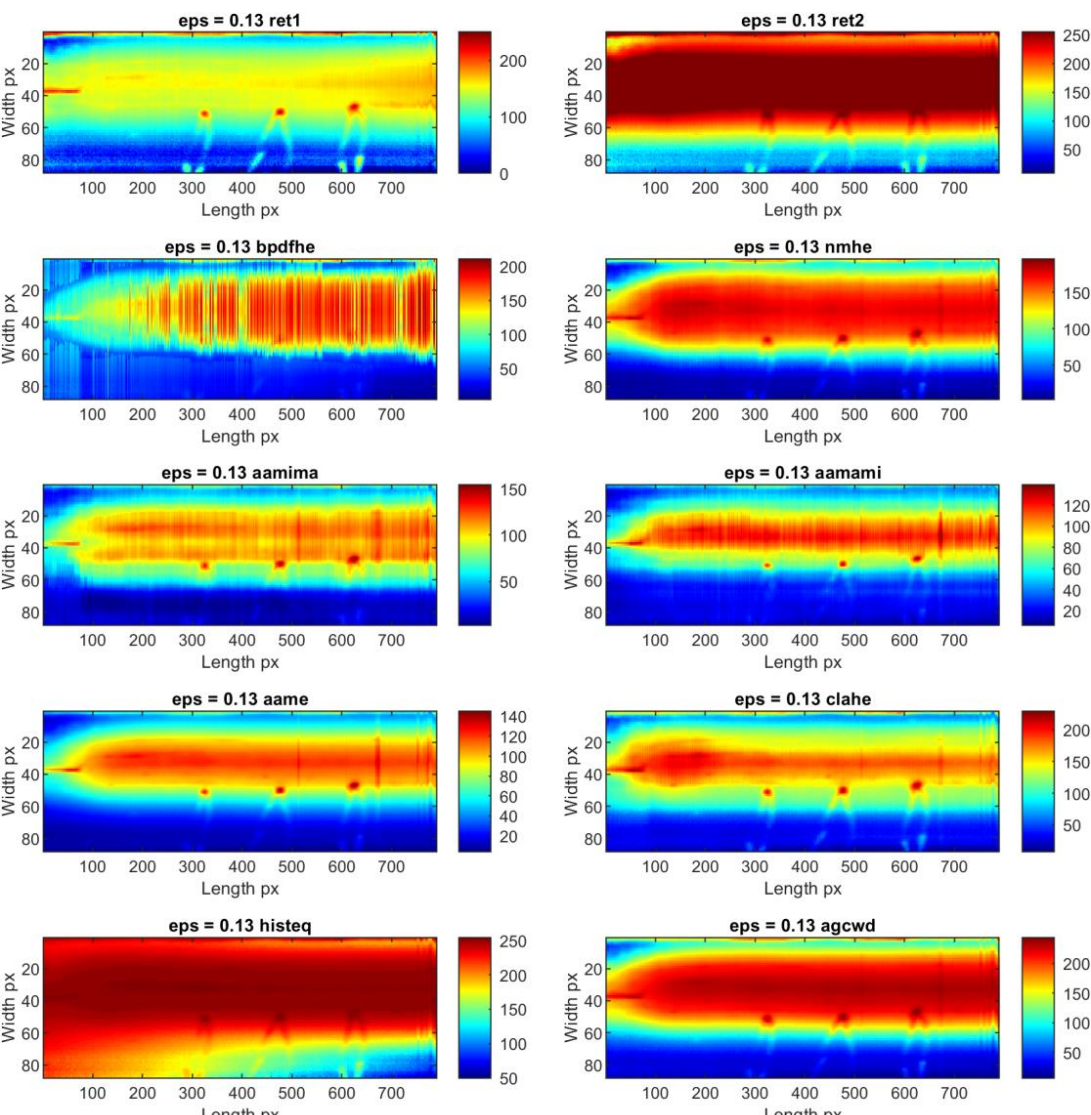
TL-reconstructed thermograms obtained for IR images taken during welding of 1.2 mm thick Inconel 625 sheets (sample ID 12.5) processed with different dynamic range compression methods.

Thermograms presenting the whole joint after the time-location transform for two exemplary sequences: ID 10.3 (Figure 12 and Figure 13) and ID 12.5 (Figure 14 and Figure 15) that provide valuable information about the welding process. TL-reconstructed thermograms suffer predominantly from the over-enhancement of background content that leads to oversaturation. Another noticeable drawback in the results is the excessive attenuation of low-contrast details, which blend with the background and are not even visible. Notice that the result brings out more details from the shadowed (hot) areas (affected with reflected temperature and hot welding torch, while maintaining good contrasts elsewhere (fine detail of hot joint and in cold base material). Slight contrasts in hot regions become easier to see. It can be seen, that similar phenomenon occur in the case of enhancement of individual thermograms.

After detailed analysis, it was found that the original reconstructed thermograms are almost identical to the results if the ‘aame’ method is used. In this case, the weld and HAZ zones are broad, and the value gap between them is low. The use of ‘clahe’ allowed us to obtain an image in which the hot central joint zone is narrower. A completely different visualization is present in the thermogram which is the result of the ‘aamima’ algorithm. There is a clear low-temperature space (joint) between two hotter HAZ areas. Unfortunately, there is high local noise in the form of significant variations in consecutive pixel columns. The presence of hotspots from the thermocouples mounted on the workpiece does not influence the results in any way and is not a source of noise.

**Figure 16:**
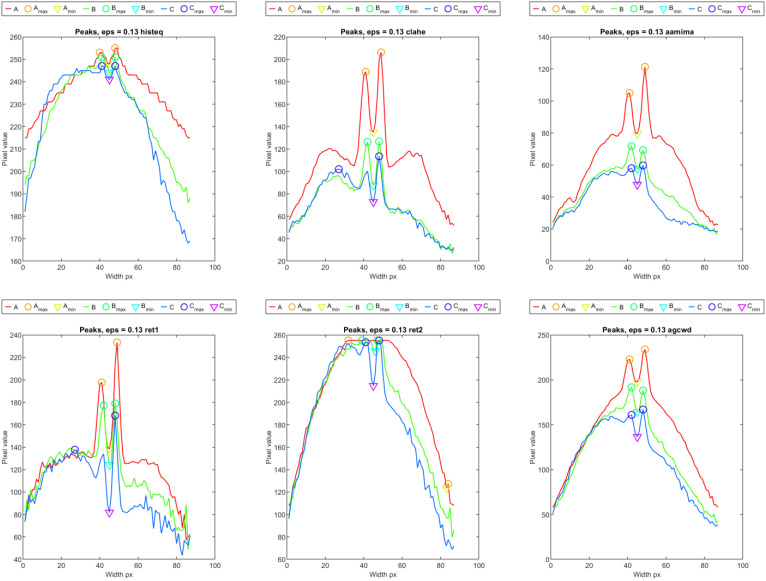
Profiles selected on TL-reconstructed thermograms with automatically chosen peaks (ID 10.3).

Cross-sections taken for the exact locations on the weld length (Figure 16) were the basis for automatic peak selection, which can be the basis for automatic weld-face width measurement. According to the results, it was found that a higher spatial resolution of thermograms is demanded to quantify minor variations in geometry that, in the data gathered during the research, are at the subpixel level.

**Figure 17:**
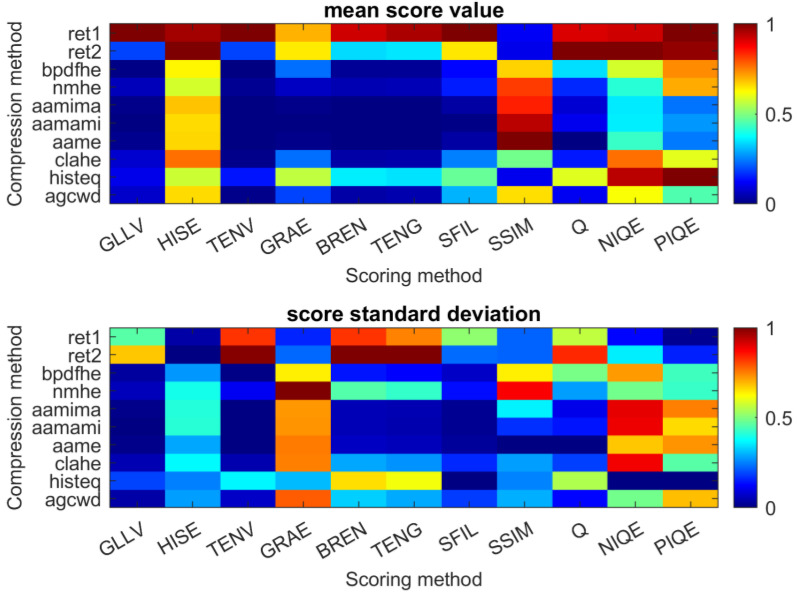
The objective metric score of different compression methods.

The results of the TL transform were evaluated in terms of thermogram quality. Different objective methods were used, including edge preservation from the original image, image sharpness, and the measuring of informational content. The results of measure values and standard deviation for individual compression methods were gathered in a graphical form in Figure 17 and Table 3. The application of objective measures was compared with the subjective evaluation of the results. This is because objective measures do not consider stability temperature changes in time/width, which can be very low for certain methods (e.g., ‘bpdfhe’). The vast majority of measures returned the highest ranking for ‘ret1′. Nevertheless, it can be seen that the high mean score is connected with a high standard deviation. Thus, the results generated with ‘ret1′ are unstable in terms of quality. Interpreting the metrics for which the reference image was required is difficult and can lead to misleading conclusions. The SSIM and Q measures were used to check the degree of similarity between the original and processed images/thermograms. In the investigated case, a high value of SSIM measurement may indicate a slight improvement in the quality of the thermogram, the result being the co-dynamic range compression because only a small amount of new details can be revealed and emphasized on the resulting thermogram in comparison to the original IR image. On the other hand, low SSIM values could result from a highly noisy image or an image with many new details that are not present in the original image.

**Figure 18:**
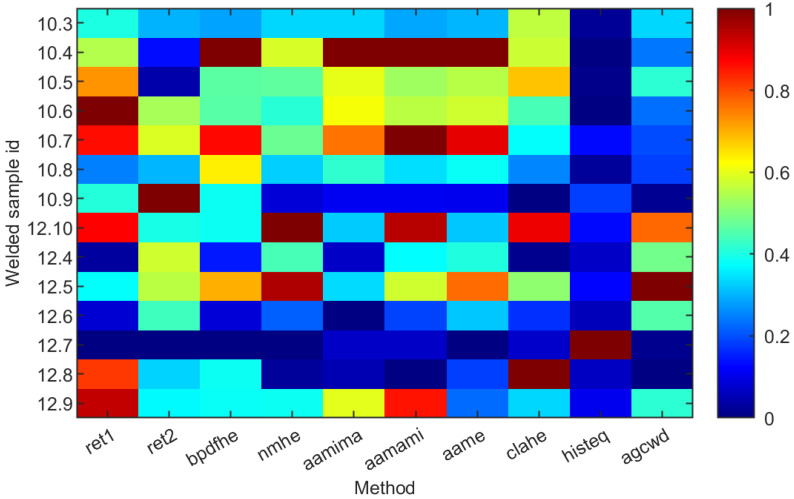
Peak-peak temperature ${\hat{T}}_{p-p}$ normalised value distribution for selected samples over various compression methods.

Normalized peak-peak temperature ${\hat{T}}_{p-p}$ was calculated for all considered welds and enhancement methods (Figure 18). It can be seen that there is a strong correlation between the application of the simple histogram equalization (‘histeq’) and the ‘sharpness’ of resulting thermograms. Additionally, method ‘ret1′, according to the ${\hat{T}}_{p-p}$ factor, objectively gives the best results compared to subjective human assessment, and it was revealed that there is a noise present that makes those thermograms impossible to use by a human operator. In contrast, in others, results are precise and not affected by noise.

**Figure 19:**
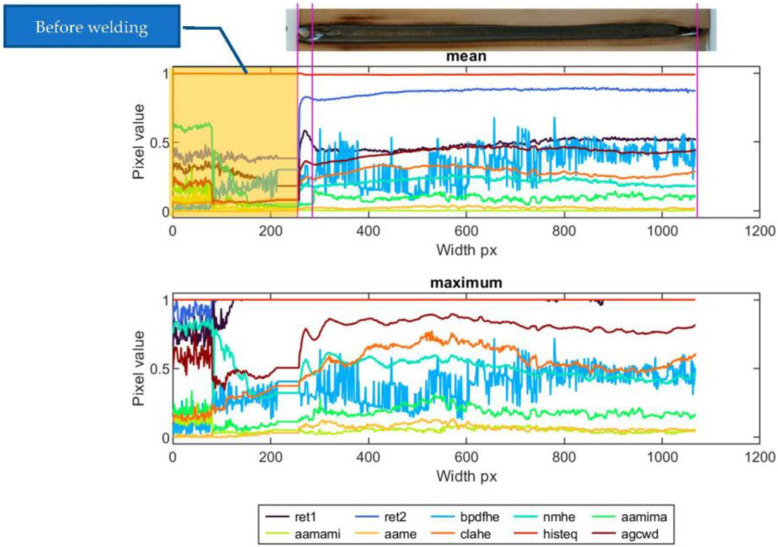
Mean and maximum horizontal face profiles of sample ID 10.8.

When assessing profiles that were calculated as mean and maximum values from weld cross-sections, it can be found that the correspondence between face condition and profile value varies significantly for different dynamic-range-compression methods. In Figure 19, there is a set of profiles calculated for sample ID 10.8. The plot consists of the part of the profile that is the result of the TL transform. Additionally, there is an apparent part representing the temperature distribution in the sample, which was acquired before the welding started and the welding machine table began to move below the welding torch. In all thermograms, this part was cropped to ease the interpretation of results. The signal is more unstable for the ‘bpdfhe’ method, for both mean and maximum profiles. Similar behavior can be observed in the remaining methods, excluding ‘ret1′ and ‘histeq’. In the first, in the short range, there is a relatively significant increase in apparent temperature, resulting from the burn trough at the beginning of welding (delayed start of the table after the welding arc started to glow). Then, as a result of lifting the sample towards the welding torch, the temperature increased, which caused the widening of the joint face and the HAZ. Then, the process was stabilized (narrowing of the welding face). In the final weld zone, another increase in joint width was observed. The described face width and quality change scheme is most evident in the profiles (mean and max) generated using the ‘aamima’, ‘agcwd’ and ‘clahe’ methods. For ‘ret1′ and ‘ret2′, changes are noticeable only in mean profiles, but the slight variation in weld-face width in the middle of joint length remains unrevealed. The ‘aame’ and ‘histeq’ methods are insensitive to changes in the geometry of the weld face.

**Figure 20:**
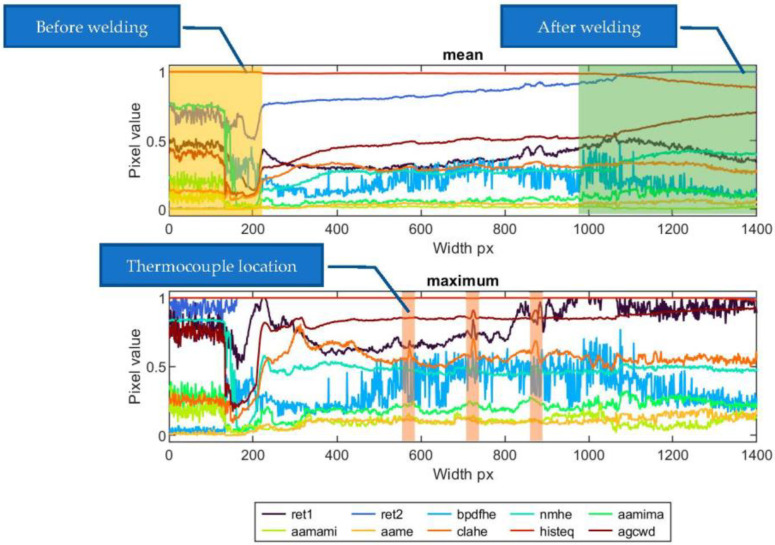
Mean and maximum horizontal face profiles of sample ID 12.5.

For sample ID 12.5 (Figure 20), the temperature distribution throughout the length of the TL-generated sample (during the entire welding process) is uniform. Instant changes in apparent temperature are related to the emissive properties of the thermocouples and wires. This is the case, especially for the maximum profile. Detailed evaluation of the results was focused on the methods of ‘aamima’, ‘agcwd’ and ‘clahe’ that subjectively gave the best results. When comparing the size of the weld face (Table 2), it can be seen that at the beginning of the joint, the weld face is broader, and the apparent temperature represented in the maximal pixel value is also higher. The ‘aamima’ method best reflects the change in geometry. Dealing with the mean parameter is an option when a global characteristic of the weld face has to be assessed, such as width or possible presence of large cracks or burn troughs. In the max feature, occurrences of local inconsistencies or faults cause small spots with higher apparent temperatures.

In the research, this type of fault (spatters, slag inclusions, etc.) was not observed or simulated, but similar conditions can be observed for spots in which thermocouples are mounted. Locations are present in the maximum feature plot for sample ID 12.5 (marked in Figure 20), where thermocouples cause an increase in apparent temperature on a narrow part of the weld length.

**Figure 21:**
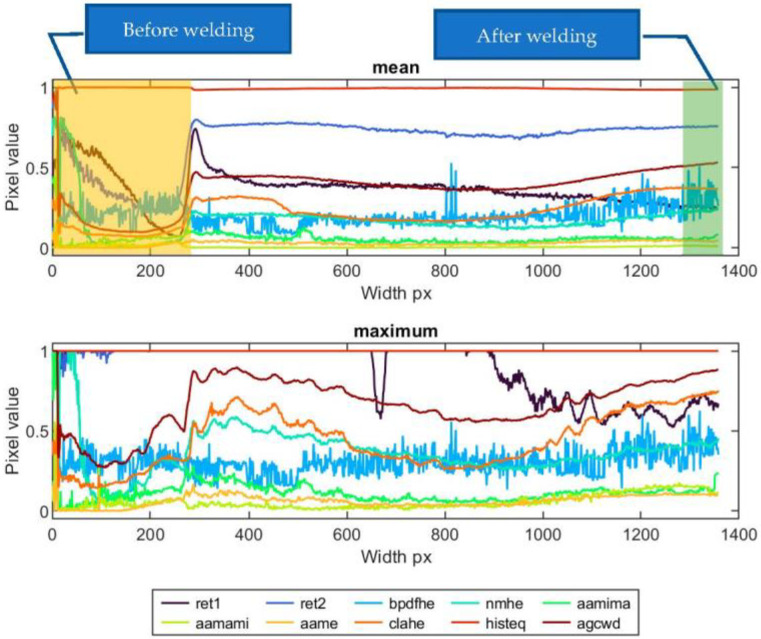
Mean and maximum horizontal face profiles of sample ID 10.3.

Similar results can be noticed for sample ID 10.3 (Figure 21). In this case, there is a weld with a narrower face width in the central part of the joint length. This change in face geometry corresponds to a decrease in the apparent temperature. For all samples and weld-face geometry anomalies, this relationship is clear.

## 4. Conclusions

The analysis of dynamic-range-compression methods for the generation of approximated infrared (IR) images is presented in the paper. The results of thermogram processing were used to quantify the quality and geometry. It was found and confirmed that methods based on local histogram estimation and multiscale wavelet-based image restoration techniques could provide new thermograms with enhanced details. A key issue for multiscale methods is choosing the right combination of merging rules that will lead to optimal output images in which the temperature contrast will be increased. That means small temperature variations in neighboring areas could be emphasized by increasing the value span between those regions, and large temperature gradients could be flattered. Nevertheless, no universal and robust method can be applied to different datasets, even if the sequences were taken under similar conditions and reflect identical phenomena.

In the paper, only methods not based on machine-learning solutions are evaluated. It is because acquiring a sufficiently numerous set of samples that can be applied for the compression of thermograms is difficult, expensive, and time-demanding. The method is needed to calculate a reference level that will be used to quantify horizontal profiles. The reference value must be estimated to be an optimal welding condition that led to the formation of a high-quality joint with the desired properties and geometry. Without reference, generated profiles could only be evaluated relatively, by calculating disturbances and changes in profile value in the time/length of the weld. Future investigations will also be connected to the elaboration of a new dynamic-range-compression method, which will consider the system of human vision and raw thermogram requirements that must be prepared for visualization using colourmaps. Other welding processes will be applied to increase the available database of thermogram sequences, including laser welding. It will allow the application of machine-learning methods, which could lead to the elaboration of general and multipurpose compression models. Those models could be merged with other ML models to provide a complex tool to process and analyze thermogram sequences to detect weld-face faults and inconsistencies unanimously.

## Figures and Tables

**Table 1:** Chemical composition of the investigated Inconel superalloys.

Super- Alloy	Element Concentration, % wt													
	Ni	Cr	Fe	Mo	Nb	Co	Mn	Cu	Al	Ti	Si	C	S	P
Inconel 625	60.7	21.76	4.27	8.96	3.56	0.07	0.07	-	0.14	0.18	0.08	0.01	0.0003	0.007

Nb + Ta—3.56%, N—0.01%.

**Table 2:** TIG welding parameters used for the generation of samples and geometrical properties of the weld face measured on all samples.

ID	Plate Thickness mm	Current A	Welding Speed mm/s	Face Width Point A mm	Face Width Point B mm	Face Width Point C mm	Thermocouples Offset mm
12.1	1.2	60	3.0	7.11	8.60	no joint	6.0
12.2	1.2	60	3.0	5.40	5.39	6.17	3.0
12.3	1.2	60	4.0	4.50	4.96	4.61	6.0
12.4	1.2	60	4.0	5.11	4.60	4.35	3.0
12.5	1.2	60	5.0	4.40	3.42	3.43	3.0
12.6	1.2	60	5.0	3.78	3.47	3.72	6.0
12.7	1.2	60	3.0	6.32	6.03	6.22	-
12.8	1.2	60	4.0	5.02	4.19	4.52	-
12.9	1.2	60	5.0	3.29	3.56	3.35	-
12.10	1.2	60	7.0	burn trough	3.14	3.09	-
10.1	1.0	35	3.0	2.77	2.21	1.99	3.0
10.2	1.0	35	3.0	1.87	1.36	1.46	6.0
10.3	1.0	45	3.0	1.54	1.87	1.37	-
10.4	1.0	45	3.0	2.14	1.87	1.92	3.0
10.5	1.0	45	3.0	1.85	2.08	2.15	6.0
10.6	1.0	40	3.0	2.01	2.37	2.94	-
10.7	1.0	35	3.0	1.71	1.68	1.70	-
10.8	1.0	30	3.0	3.45	3.11	3.18	-
10.9	1.0	25	3.0	2.83	2.39	2.34	-

**Table 3:** The objective metric score of different compression methods.

	GLLV	HISE	TENV	GRAE	TENG	SIFL	SSIM	Q	NIQE	PIQE
ret1	1.00 (0.46)	0.96 (0.03)	1.00 (0.82)	0.69 (0.15)	0.92 (0.82)	0.95 (0.74)	1.00 (0.51)	0.11 (0.22)	0.90 (0.56)	0.91 (0.12)
ret2	0.18 (0.67)	1.00 (0.00)	0.18 (0.99)	0.64 (0.22)	0.33 (1.00)	0.34 (1.00)	0.64 (0.22)	0.10 (0.22)	1.00 (0.83)	1.00 (0.35)
bpdfhe	0.00 (0.03)	0.62 (0.27)	0.00 (0.00)	0.23 (0.64)	0.02 (0.14)	0.02 (0.12)	0.12 (0.06)	0.66 (0.63)	0.34 (0.49)	0.57 (0.71)
nmhe	0.05 (0.05)	0.57 (0.39)	0.01 (0.11)	0.06 (1.00)	0.04 (0.45)	0.05 (0.42)	0.15 (0.13)	0.81 (0.87)	0.16 (0.27)	0.41 (0.48)
aamima	0.01 (0.01)	0.68 (0.40)	0.00 (0.00)	0.01 (0.72)	0.00 (0.05)	0.00 (0.04)	0.03 (0.01)	0.84 (0.36)	0.08 (0.10)	0.35 (0.89)
aamami	0.00 (0.00)	0.65 (0.41)	0.00 (0.00)	0.00 (0.72)	0.00 (0.05)	0.00 (0.04)	0.00 (0.01)	0.93 (0.16)	0.10 (0.14)	0.35 (0.88)
aame	0.01 (0.01)	0.66 (0.28)	0.00 (0.00)	0.00 (0.75)	0.00 (0.06)	0.00 (0.05)	0.03 (0.02)	1.00 (0.00)	0.00 (1.52)	0.42 (0.67)
clahe	0.07 (0.04)	0.76 (0.37)	0.00 (0.04)	0.23 (0.74)	0.03 (0.28)	0.04 (0.26)	0.24 (0.16)	0.48 (0.27)	0.14 (0.18)	0.76 (0.88)
histeq	0.10 (0.19)	0.57 (0.24)	0.14 (0.36)	0.56 (0.30)	0.35 (0.65)	0.34 (0.61)	0.47 (0.00)	0.10 (0.25)	0.59 (0.54)	0.93 (0.00)
agcwd	0.07 (0.03)	0.65 (0.28)	0.01 (0.06)	0.18 (0.78)	0.03 (0.32)	0.04 (0.29)	0.30 (0.18)	0.65 (0.29)	0.11 (0.12)	0.62 (0.48)

## Data Availability

The data presented in this study are available upon request from the corresponding author.
